# The Diagnosis as a Guide for a Life Trajectory: An Autobiographical Case Report

**DOI:** 10.7759/cureus.19466

**Published:** 2021-11-11

**Authors:** Carolina Monte Santo Burdman Pereira, Felipe Monte Santo, Carlos Alberto Bhering

**Affiliations:** 1 Pediatrics, Vassouras University, Rio de Janeiro, BRA; 2 Neurosurgery, Sankt Gertrauden-Krankenhaus, Berlin, DEU

**Keywords:** patients, self report, empathy, physician-patient relations, autobiographical case report

## Abstract

In surgical procedures in the pediatric population, the doctor-patient relationship becomes more complex, extending to the doctor-patient-family relationship. This case report presents my perspective as a pediatric patient with the diagnosis and surgical excision of a cervical lymphangioma and later a hemangioma, demonstrating the impacts and repercussions on my life trajectory. The quality of medical services depends on the relationship between professionals and the user. Thus, medical care can no longer be organized considering only the effectiveness. It is the doctor's responsibility to understand and manage his attitudes so that all patients have individualized care worthy of a life trajectory with resilience. By doing this, who knows, maybe we are generating a great stream of future doctors who can better understand their patients?

## Introduction

The clinical encounter between a patient and a physician is the essence of medical care [1]. Within an increasingly commercialized and globalized medical industry of modern health care, empathy has been a target of discussion in scientific studies due to the enormous fear of distancing and detachment of physicians in relation to their patients [2].

There are numerous definitions in the literature of the term "empathy." An extensive review of the literature by Morse et al. summarizes empathy in four dimensions, describing it as an emotional act (a subjective experience of another person's state), a personality or moral dimension (practice motivated by altruism), a cognitive act (the ability to identify and understand the other), and as a sphere of behavior (a way of communicating one's understanding) [3].

In the history of medicine, it is well known that the quality of the doctor-patient relationship has a significant impact on therapeutics. Empathy is certainly an essential ingredient of this relationship [4]. In this regard, studies on the doctor-patient relationship have been highlighted in the medical literature, either by the presence of the patient narrative as a therapeutic tool or as a model for improving communication techniques that provide better quality in this relationship [5].

In surgical procedures performed in the pediatric population, the physician-patient relationship becomes even more complex, extending to a physician-patient-family relationship. There is also the concern with the future development and proper growth of the pediatric patient, requiring greater sensitivity in conducting the entire clinical-surgical care. Thus, it requires a different approach in the communication of the diagnosis, bad news, and therapies involved, which may contribute to the construction or limitation of an appropriate bond, user satisfaction, adherence to treatment, and continuity of the child's healthy life trajectory [5]. In a study conducted by the American Academy of Orthopaedic Surgeons, 75% of orthopedic surgeons believed that their communication with patients was satisfactory, while only 21% of patients agreed with this claim. Barriers to effective and successful communication include fear of physical or verbal abuse, excessive physician workload, unrealistic patient expectations, as well as fear of litigation and anxiety [6].

This case report presents my perspective as a pediatric patient with the diagnosis and surgical excision of a cervical lymphangioma and later a hemangioma, demonstrating the impacts and repercussions on my life trajectory. My goal in describing this report is to provide a resource for professional colleagues regarding the doctor-patient relationship and the development of soft skills - attitudes and behaviors that can contribute to better medical practice.

## Case presentation

At the age of three years, I was diagnosed with a lymphangioma in the cervical region. Five years later, at the age of eight, I was diagnosed with a hemangioma in the same anatomical region. Initially, the first contact I had with the health service was through my pediatrician, who accompanied me regularly. After that, I was referred to the head and neck surgeon to continue the investigation. Through consultations and complementary exams, the diagnosis was quickly made and the procedure was promptly planned, with surgery scheduled for next month.

The surgery was performed in a private hospital and was partially reimbursed by the health plan. Even with the health insurance, during the whole process, there was a great financial impact on my family, mostly because the complementary exams and procedures were done with an urgency that was not compatible with our financial situation. Besides, resorting to the public health system would imply a delay that could probably cause complications. The surgeon was available for all questions, always attending with patience, cordiality, and even facilitating the whole bureaucratic process for reimbursement from the health plan. Moreover, although my parents were emotionally vulnerable, they were never sad or desperate in front of me. On the contrary, they always handled the situation with lightness and strength that always conveyed courage and the positivity that what I was going through would have little effect on my life. If I can cite one benefit of having gone through all this as a child, I would cite the naivety of not knowing the seriousness of my condition. My parents took advantage of my ignorance and turned this into resilience, always showing me that surgery was an obstacle that would be quickly resolved.

The greatest reassurance throughout the process was the speed between diagnosis and surgery (the potential cure), especially because the tumor distorted the aesthetics and facial anatomy, making it difficult to have contact with other children of the same age. My mother tells me that every weekend we went to a park to play in the morning. When I showed up at the park with the lymphangioma occupying part of my face, some mothers stopped their children from playing with me, claiming that it was not safe. My mother says that at that time she never felt so angry in her life, but she just told them that it was nothing contagious and we never went back there. I am sure it was very hard for her not to cry at that moment. Nowadays I wonder how many stares my mother got when she was walking down the street with me, all because of a benign tumor that distorted my appearance.

The histological reports showed benignity and absence of atypia. The procedure for both cases was hospitalization with surgical excision, due to the close relationship with the left carotid artery, and biopsy. The surgical procedures were uneventful. The final result was a single, almost imperceptible, scar. Afterward, ultrasonographic follow-up exams were performed for four years to exclude possible relapses and the need for a new surgical procedure. Fortunately, no recurrences were detected. The memories I keep of my two hospitalizations were of extreme importance for my future, as well as my family members who accompanied the entire process. Much of what I remember encompasses only the surgeon who operated on me, although I know it was the work of a team that got me here. I do not know if this means that most communication was always with him, but my parents always mention how serious, kind, and concerned he was about me. Unfortunately, until today, I have found only a few professionals with these characteristics and I am finally beginning to understand why his figure marked my parents and me so much.

The welcome from the whole team in promptly meeting the needs of such a delicate moment was extremely important and fundamental to awaken in me a new love, medicine.

## Discussion

According to patients, the quality of medical services depends 30-40% on the diagnostic and therapeutic ability of the physician and 40-50% on the relationship between the professionals and the patient. The debate about the doctor-patient relationship and its short- and long-term implications becomes a guide for current and future medical conduct. Thus, it is no longer possible to organize medical care considering only the effectiveness in curing a disease but to take into account the respect for the patient's subjective values, the promotion of the patient's autonomy, and the protection of cultural and individual diversities [5].

For the first surgery, I was hospitalized on the eve of my fourth birthday. A date that commemorates vitality and the construction of plans for the future quickly became a moment of great tension and fear for me, my parents, and my family. An essential point of comfort during this moment was seeing the figure of the doctor and the health team actively participating in my personal celebration in a cordial and professional manner, making themselves present beyond the clinical setting. In addition, the organization of the medical service, with visiting hours before and after the surgical procedure, was able to emphasize and respect my autonomy and integrality. In this way, I was able to seek comfort from friends, family members, and other acquaintances in an attempt to alleviate the anxiety imposed by the pathology and the surgical procedure.

The scientific literature shows that doctors who have empathy and demonstrate it can provide better care to their patients [2]. Furthermore, a physician who provides more humane care has higher levels of effectiveness. There is already evidence that patients can identify and sense empathic behavior and exhibit measurable physiological responses [2]. As an example, patients with influenza who perceived empathic behavior from their physicians showed changes in their immune system and a reduction in the duration and severity of their symptoms. Diabetic patients showed better control of their blood glucose and cholesterol levels than patients with non-empathic behavior [2,4]. In oncology, patients were able to show greater satisfaction, decreased emotional stress, and increased self-confidence [2].

Looking at parents as experts in the care of their children, they should be incorporated into shared decision-making in a culturally appropriate way. The development of assertive communication by the physician and his team enhances their professionalization. Thus, it allows the use of affective-emotional survival in family members that contributes to therapeutic adherence and patient longitudinality (lifelong medical follow-up) [7]. Diagnosis can also be considered an important tool available to the physician since no diagnosis occurs without emotional repercussions. It is in this context that diagnosis presents itself as a spectrum of possibilities - a therapeutic experience, a relief for the patient and his family, a new path, a traumatic experience, and even a death sentence [8].

It is important to emphasize that a diagnosis generates vital consequences in a family dynamic, and can even be a threat to its homeostasis. When we talk about the pediatric age group, the family becomes an important instrument to provide support and collaborate with the necessary treatment. This support extends throughout the patient's childhood, often impacting the child's development. This same line of reasoning applies to the physician and his conduct toward the pediatric patient, even more so if it is a potentially life-threatening disease such as cancer.

A striking point of the doctor-patient relationship in my clinical case was the explicit concern invested in me, something reported by the parents as key to establishing trust from the beginning of the doctor-patient-family relationship. A welcoming and calm environment, together with consultations under a patient, calm, and accessible to the layman's understanding discourse was essential to ensure the safety of the family members. The resolution of the pathology transits not only in the cure but also in a long-term follow-up.

After the excision of the tumors, I went through numerous imaging follow-ups over the years, in addition to consultations to check the anatomical, physical, and also mental integrity, especially since I was a developing child at the time. The bond established was built through a complete follow-up, based on equality, respecting not only me, but my family in all humanistic dimensions, going against the paternalistic and outdated concept of doctor-patient relationships, often currently present.

According to Beauchamp and Childress, "There is, in medicine, a temptation to use the authority of the physician's role to foster or perpetuate the dependence of patients, instead of promoting their autonomy. Fulfilling the obligation to respect the patient's autonomy, however, requires enabling the patient to overcome his or her sense of dependence and to obtain as much control as possible or as much control as he or she desires" [9]. It is possible to say that although I faced two frightening diagnoses and that certainly influenced my family stability, I took from this process a great resilience to look to my future with more certainty and sensibility. Much of this resilience came from a consolidated communication and relationship with all the family links through the medical team.

Throughout medical school, students' optimism and sensitivity often morph into an essentially clinical view of the specialty and are reinforced during the clinical phase and rotations. In a study by Lorenz et al., third- and fourth-year medical students were excused from their traditional clinical activities to follow pediatric cancer patients, maintaining daily contact with their narrative of observations and perceptions. Coupled with a literature review, the study identified the development of a rich understanding of patient experiences, with greater humanistic appreciation and reflection of the full context of the disease, including that presented by the children themselves. In the end, most participants found that they had rediscovered and rekindled their intrinsic empathic behaviors and believed they would continue such insight in future rotations [10].

In the need to maintain the tripod of beneficence, non-maleficence, and autonomy, a diagnosis must be presented correctly, because it is capable of provoking different forms of reactions and emotions. The big issue is whether these emotional repercussions will be positive or negative, in which the physician plays a crucial role in this dynamic. Similar to the participants in the aforementioned study, I was able to identify an example of the cycle of caring and being cared for, understanding with greater sensitivity that empathy, honesty, and humanity should take first place in medicine. With that, I entered medical school in 2016.

The hidden curriculum is the term used to identify the fruit of interpersonal relationships experienced academically that exceed the formal or traditional curriculum, with a focus on those that arise from everyday situations [11]. Its characteristics include the doctor-patient-family relationship, empathy, communication of bad news, among others [12]. When the physician has already put himself in the place of a patient in need of care, it is more natural to develop and understand such characteristics. These components, therefore, become fundamental as future professionals learn to go through challenging and emotional experiences that are routine in medical practice [13].

Medicine is a great example that should be passed on to each patient in the same way that we would like it to be with ourselves. A phrase that sums up this non-traditional case report, from my perspective as an author-patient-future physician, is that, as Isaac Newton stated, "If I have seen further, it is by standing under the shoulder of giants." Therefore, today I write this article understanding that to graduate as a doctor, it was essential to have as an example a doctor who saw me beyond a patient and who touched my soul in a moment of apprehension, fear, anxiety, and fear. The beauty of medicine lies in setting an example, and today I am the living example that a doctor-patient relationship makes a difference because I am committed to touching people's souls in the same way that mine was touched during the two surgical procedures.

## Conclusions

Although often underestimated, the way in which the diagnosis or disease is communicated represents a crucial part of a patient's life trajectory and may contribute to resilience or a traumatic experience. When talking about neoplasms, benign or malignant, the picture extends with greater complexity, and the physician must adopt a central position in the tripod autonomy, beneficence, and non-maleficence. Whether in a child with benign cervical neoplasia or not, the physician and his team must understand that the therapeutic process involves not only the cure but the ability to see the patient in his entirety, regardless of age group, as exemplified in the case report above.

A diagnosis, a report, or a therapy can change a life, positively or negatively. It is the doctor's responsibility to understand and manage his attitudes so that all patients have individualized care worthy of a life trajectory with resilience, meaning, and care. With my example of attention, welcoming, and caring, I was able to awaken a new love, medicine, bringing to my future profession a new look at empathy and the emotions of my patients. By doing this, who knows, maybe we are generating a great stream of future better doctors who can better understand their patients?
